# Application of Swarm Intelligence Optimization Algorithms in Image Processing: A Comprehensive Review of Analysis, Synthesis, and Optimization

**DOI:** 10.3390/biomimetics8020235

**Published:** 2023-06-03

**Authors:** Minghai Xu, Li Cao, Dongwan Lu, Zhongyi Hu, Yinggao Yue

**Affiliations:** 1 School of Intelligent Manufacturing and Electronic Engineering, Wenzhou University of Technology, Wenzhou 325035, China; xmhemail@126.com (M.X.); caoli198723@163.com (L.C.); 2 Intelligent Information Systems Institute, Wenzhou University, Wenzhou 325035, China; 21451943016@stu.wzu.edu.cn (D.L.); hujunyi@163.com (Z.H.)

**Keywords:** swarm intelligence optimization algorithm, image processing, image segmentation, image features, edge detection

## Abstract

Image processing technology has always been a hot and difficult topic in the field of artificial intelligence. With the rise and development of machine learning and deep learning methods, swarm intelligence algorithms have become a hot research direction, and combining image processing technology with swarm intelligence algorithms has become a new and effective improvement method. Swarm intelligence algorithm refers to an intelligent computing method formed by simulating the evolutionary laws, behavior characteristics, and thinking patterns of insects, birds, natural phenomena, and other biological populations. It has efficient and parallel global optimization capabilities and strong optimization performance. In this paper, the ant colony algorithm, particle swarm optimization algorithm, sparrow search algorithm, bat algorithm, thimble colony algorithm, and other swarm intelligent optimization algorithms are deeply studied. The model, features, improvement strategies, and application fields of the algorithm in image processing, such as image segmentation, image matching, image classification, image feature extraction, and image edge detection, are comprehensively reviewed. The theoretical research, improvement strategies, and application research of image processing are comprehensively analyzed and compared. Combined with the current literature, the improvement methods of the above algorithms and the comprehensive improvement and application of image processing technology are analyzed and summarized. The representative algorithms of the swarm intelligence algorithm combined with image segmentation technology are extracted for list analysis and summary. Then, the unified framework, common characteristics, different differences of the swarm intelligence algorithm are summarized, existing problems are raised, and finally, the future trend is projected.

## 1. Introduction

With the development of science and technology, images have been used in all aspects of people’s lives in recent years, such as mobile phone photography, license plate recognition, face recognition, and web page image material. In-depth research on images can improve people’s living environment, change people’s way of life to a large extent, and improve people’s quality of life [1]. Image processing involves a wide range of fields, including image transformation, restoration, segmentation, enhancement, matching, and classification. Image processing technology mainly includes image enhancement and restoration, image compression and coding, image segmentation, image matching, and image classification, among which many problems need to be optimized in the fields of image restoration, image segmentation, and image distribution. For example, search for the best matching block in image restoration, find the best segmentation threshold in image segmentation, and find the best matching position in image matching [2]. When using an ordinary global traversal algorithm to solve these problems, the calculation time may reach an unacceptable level. Therefore, intelligent algorithms can be applied to optimize these problems for more efficient solutions.

The advantages of applying artificial intelligence algorithms in image processing work mainly reflect the following points [3]. First, it can effectively replace traditional manual work, and while improving work quality and efficiency, it also liberates labor costs. Artificial intelligence can work endlessly at high speed and promote the improvement of image processing technology’s quality and efficiency. Second, the use of artificial intelligence algorithms in image processing can break through the inadequacy of image segmentation and image recognition in traditional image processing and promote the accuracy and value of image processing [4]. Third, the use of artificial intelligence algorithms can preprocess data and fully demonstrate the value of stored information. At the same time, it can also maximize the functions of image recognition, storage, and self-analysis, providing strong support for efficient image processing in the future.

### 1.1. Problem Description and Research Motivation

With the rapid development of digital image processing technology, digital image processing is becoming more widely used in military, medical, industrial production, telemetry, remote control, and other fields. The complexity and diversity of image information features are becoming more and more obvious, and the processing of image information is becoming more and more difficult. The uncertainty of image information and the difficulty of modeling make traditional optimization methods powerless when solving complex image processing problems. Therefore, complex image processing technology has increasingly urgent requirements for efficient intelligent computing and optimization technology [5].

From the perspective of biology, the bionic intelligent optimization algorithm developed in recent years, according to the characteristics of organisms in a certain environment through the process of self-organization to continuously adapt to environmental changes, makes the optimization algorithm have strong adaptive characteristics, ensuring that the algorithm is relatively stable. Good fault tolerance and robustness. In addition, in the bionic intelligent optimization algorithm, the learning ability of organisms that can gain experience from themselves and the group is used to share information and guide each other in the optimization process, which improves the convergence speed of the algorithm. Under a certain probability, individuals in the algorithm can find the optimal results without the need for special problem information according to pre-set simple rules. Through biological self-organization ability, adaptive behavior, and information sharing mechanisms, they can achieve efficient optimization performance. When dealing with large-scale optimization problems that are difficult to solve by traditional mathematical programming methods, bionic intelligent optimization algorithms can obtain optimal or suboptimal solutions in a short time. It has also achieved good results on optimization problems. Therefore, bionic intelligent optimization algorithms have gradually become a research hotspot in the field of modern optimization algorithms. How to use the powerful optimization ability of the bionic intelligent optimization algorithm to perform complex image processing is an urgent problem to be solved [6].

For optimization problems, most methods are used to establish the objective function. For example, in the image threshold segmentation algorithm, an objective function is defined according to a specific target task within the scope of constraints, and the threshold for image segmentation is the global optimal solution of the objective function within the scope of constraints. To deal with the image segmentation problem from the viewpoint of combinatorial optimization, it mainly transforms various requirements and constraints representing image segmentation into an objective function and then transforms the image segmentation into an optimal solution process for the objective function. Therefore, it is completely feasible to use intelligent algorithms to solve the problem of image segmentation. With the continuous development of science and technology, the environment in which image processing is located has become more and more complex. This is because traditional iterative optimization algorithms have insurmountable problems such as low solution efficiency and one-sided search. At the same time, using traditional optimization algorithms to solve problems in image processing has become increasingly difficult to meet the demand for speedy solutions. Therefore, there is an urgent need to use intelligent algorithms with better optimization performance. At the same time, since image processing can be regarded as a process of solving complex nonlinear problems, intelligent algorithms can still achieve good optimization results for complex problems that are difficult to solve with traditional algorithms. Therefore, the application of the swarm intelligence algorithm in image processing has broad prospects for development.

### 1.2. Contribution

It should be pointed out that there is a lot of research work on image processing methods based on swarm intelligence optimization algorithms in academic and industrial circles at home and abroad, but this work is relatively scattered. This paper mainly systematically combs the research and key points of image processing, discusses and analyzes the research hotspots and difficulties of image processing based on the swarm intelligence optimization algorithm as a whole, and provides some references for academia and relevant practitioners.

The main contributions of our work in this paper can be summarized as follows:

(1) Describe and summarize the current popular swarm intelligence optimization algorithms in detail;
(2) In-depth analysis of image segmentation, image matching, image classification, image feature extraction, and image edge detection in the image processing diagram is carried out, and a summary is made;
(3) For five intelligent optimization algorithms such as the ant colony algorithm, particle swarm algorithm, sparrow search algorithm, bat algorithm, and salp swarm algorithm in image processing such as image segmentation, image matching, image classification, image feature extraction, and image edge detection. The applications in the paper are summarized, and their advantages and disadvantages are compared;
(4) The key indicators of image processing, such as image segmentation, image matching, image classification, image feature extraction, and image edge detection, are comprehensively analyzed and compared.

The application of intelligent optimization algorithms such as the ant colony algorithm, particle swarm optimization algorithm, sparrow search algorithm, bat algorithm, and salp swarm algorithm in image processing such as image segmentation, image matching, image classification, image feature extraction, and image edge detection is described in detail. Section 3 is the comprehensive analysis and summary of the algorithm, which combines the five swarm intelligent optimization algorithms with image segmentation, image matching, image classification, image feature extraction, image edge detection, etc., for list analysis and summary. Section 4 summarizes the full text and looks forward.

## 2. Overview of Swarm Intelligence Optimization Algorithms

**Figure 1:**
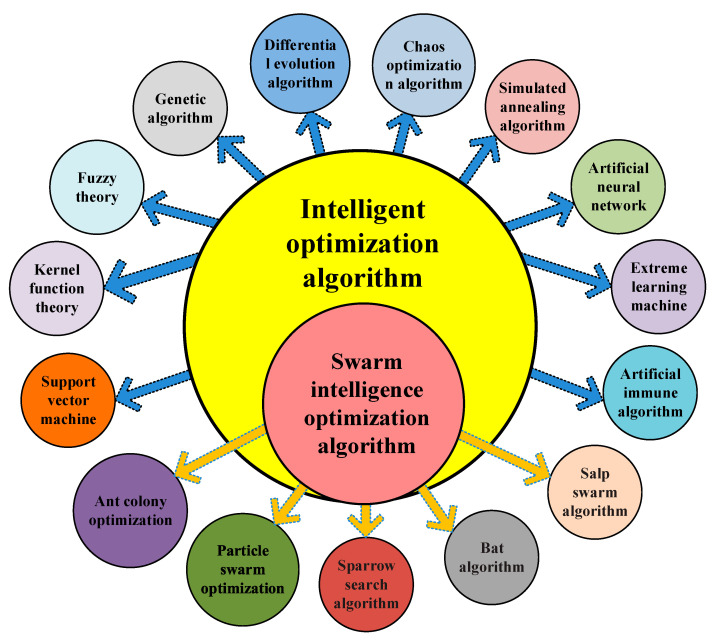
The framework diagram of the swarm intelligence optimization algorithm.

Inspired by the cooperative and competitive behavior of gregarious organisms, a new evolutionary computational technique called the swarm intelligence bionic optimization algorithm has been explored. Its application areas include multi-objective optimization, data classification, data clustering, pattern recognition, telecommunication service quality management, biological system modeling, process planning, signal processing, robot control, decision support, simulation, system identification, and so on. The swarm intelligence bionic optimization algorithm is an emerging optimization calculation method. The swarm intelligence optimization algorithm is a heuristic search algorithm that optimizes a given target based on group behavior. The framework diagram of the swarm intelligence optimization algorithm is shown in Figure 1.

The main features of the swarm intelligence algorithm are: (1) Strong robustness. Because the individuals in the group are distributed and there is no centralized control, even if an individual fails, it will not affect the group’s solution to the problem; that is, it will not affect the overall situation. (2) Simple and easy to implement. The actions that each individual can perform are simple. (3) The scalability is good. The amount of information that each individual can perceive is limited. (4) Strong self-organization. The complex behavior exhibited by the group is the result of individual interaction. (5) It has potential parallelism and distribution characteristics. Swarm intelligence algorithms can be roughly divided into three categories according to the source of inspiration:

(1) It is derived from the simple foraging behavior of animals. For example, the Shuffled Frog Leaping Algorithm (SFLA) [7] proposed by Eusuff et al. in 2003 and the Whale Optimization Algorithm (WOA) [8] proposed by Mirjalili et al. in 2016;
(2) It stems from the pure social behavior of biological populations, such as the artificial bee colony algorithm (ABC) proposed by Karaboga in 2005 [9] and the firefly algorithm (FA) proposed by Yang in 2008 [10]. The cuckoo search (CS) method proposed in 2009 [11] and the Mayfly Algorithm (MA) proposed by Konstantinos et al. in 2020 [12];
(3) Social behavior and foraging behavior derived from biological populations, such as the Bacterial Foraging Optimization (BFO) proposed by Passino in 2002 [13], the Bat Algorithm (BA) proposed by Yang in 2010 [14], and the Sparrow Search Algorithm (SSA) proposed by Xue et al. in 2020 [15].

Swarm intelligence originated from the study of ant colonies, migratory bird flocks, and other social group cooperative behaviors. By studying the group behaviors of a large number of individuals, the group behaviors are modeled, rules are created, and algorithms are proposed, which are finally used to solve practical problems. Swarm intelligence algorithms can be divided into two types: one is the collective intelligence emerging from a group of simple agents, represented by ant colony optimization algorithms. The other is to regard members of the group as particles, not agents, represented by PSO algorithms. According to this conclusion, this paper selects the ant colony algorithm and particle swarm algorithm to introduce [16].

In the combination of the swarm intelligence algorithm and traditional image segmentation technology, researchers have conducted a comprehensive review of image segmentation techniques as early as the ant colony algorithm. The literature [17] formally integrated the ant colony algorithm into the image segmentation, and another example was the particle swarm algorithm. The literature [18] optimized it to solve the medical image problem. The literature [19] designed a multi-threshold image segmentation method through the PSO algorithm, which further improved the segmentation speed under the premise of ensuring the accuracy of image segmentation results.

## 3. Swarm Intelligence and Its Application in Image Processing

### 3.1. Principles of Ant Colony Algorithm

The ant colony optimization algorithm (ACO) is a new heuristic optimization algorithm based on population optimization proposed by people affected by the feeding behavior of ants.

The mathematical model of the traditional ant colony algorithm is as follows: in the path search process, the ant will transfer information between nodes according to the number of pheromones on the path and the distance. The probability of the state transition of ant *k* from node *i* to node *j* at the *t* moment is shown in Equation (1).

(1) $$p_{ij}^{k}(t)=\{\begin{array}{ll} \frac{{\left[\tau_{ij}(t)\right]}^{\alpha }\cdot {\left[\eta_{ij}(t)\right]}^{\beta }}{\sum_{s\subset allowed_{k}}{\left[\tau_{is}(t)\right]}^{\alpha }\cdot {\left[\eta_{is}(t)\right]}^{\beta }} & ,\;if\;j\in allowed_{k}\\ 0 & ,\;otherwise \end{array}$$

where $\tau_{ij}(t)$ represents the amount of information on the (i,j) of the *t*-time road section, and the amount of information on each path at the initial moment is equal, that is, $\tau_{is}(0)=cons\tan t$; $\eta_{is}(t)$ is a heuristic function, and in the traditional ant colony algorithm $\eta_{is}(t)=1/d_{ij}$; $d_{ij}$ represents the length of the road segment (i,j).${\mathrm{allowed}}_{k}$ represents a selectable set of nodes for ants, *α* information heuristics that represent the relative importance of the trajectory. *β* expectation heuristics that represent the relative importance of visibility.

In addition, too many pheromones that remain on the path traveled by the ant will drown out the influence of the heuristic breath, so the pheromones need to be updated during the ant’s travels. Its update rules are shown in Equation (2).

(2) $$\begin{array}{l} \tau (t+n)=(1-\rho )\cdot \tau_{ij}(t)+\Delta \tau_{ij}(t)\\ \Delta \tau_{ij}(t)=\sum_{k=1}^{m}\Delta \tau_{ij}^{k}(t) \end{array}$$

where *ρ* represents the pheromone volatilization coefficient and *ρ* is valued in the range ρ∈(0,1).

Use the global pheromone update rules shown in Equation (3).

(3) $$\Delta \tau_{ij}^{k}(t)=\{\begin{array}{c} \frac{Q}{L_{k}},\mathrm{if}\;\mathrm{the}\;k-\mathrm{th}\;\mathrm{ant}\;\mathrm{passes}\;\mathrm{through}\;(i,j)\;\mathrm{in}\;\mathrm{this}\;\mathrm{cycle}\\ 0,\;\;otherwise \end{array}$$

In the formula, *Q* represents the total amount of pheromones that the ant cycles for one week and, to some extent, affects the convergence speed of the algorithm; $L_{k}$ represents the length of the road section traveled by the ant *k* in this cycle.

### 3.2. Improvement of the ACO Algorithm and Its Research Status

The ant colony algorithm is an intelligent optimization algorithm proposed by Italian scholar Dorigo et al. to simulate ant foraging behavior, which has strong robustness and adaptability. Since the ant colony optimization algorithm was proposed, scholars have continuously improved and optimized it, and most of the improvement methods are to strengthen the search capability of the ant colony. The difference lies in the direction of improving the search control strategy, and the local search methods from other algorithms are added to the original ant colony algorithm so that the ant colony algorithm can obtain a better solution [20].

To improve the search rate and avoid premature convergence of the algorithm, one must improve the ant colony algorithm itself. For example: ant colony algorithm with elite strategy [21], ant colony algorithm with reconnaissance characteristics [22], max-min ant system [23], and other algorithms. The ant colony algorithm with elite strategy mainly marks the ants on the shortest path as “elite” when the algorithm is running and enhances the pheromone on the path. Common ants perform basic ant colony search work, while scout ants use the path evaluation model to calculate the mutation probability of each line of the current optimal solution and continue to search for the optimal solution according to the mutation probability. The max-min ant system has three improvements [24]. In addition, ref. [25] adjusts the search direction of each stage by adding a dynamic search induction operator and using a decay model to speed up the convergence speed and improve the quality of the solution.

The combination of the ant colony algorithm and other algorithms is also an important optimization method. Ref. [26] combines the ant colony algorithm with the artificial potential field algorithm and uses the artificial potential field method in the initial pheromone allocation of the ant colony algorithm to reduce the problem of falling into a local optimum. In addition, to improve the state transition function of the ant colony algorithm, the potential field guidance function is introduced, which reduces the possibility of blind selection during the search process and reduces the overall search time. To solve the problem of UAV conflict, the literature [27] added a speed adjustment strategy to the original ant colony algorithm, designed a solution combined with the heading adjustment strategy, and achieved good results. To improve the coverage and reliability of the information perception layer of the power Internet of Things, the literature [28] completed the networking process of CPW by combining Brownian motion with local convergence number control and the ant colony algorithm, and combined with the original technology, the cross-layer integration of low-voltage power lines and micro-power wireless communication networks was completed.

### 3.3. Improvement and Application of ACO in Image Processing

(1) Image segmentation

In the application of the ant colony algorithm in the field of image segmentation, the improvement can be divided into three categories.

In the field of image segmentation, some literature has introduced the ant colony algorithm to improve the problems of traditional image segmentation algorithms, such as long segmentation times and low segmentation accuracy. The traditional watershed segmentation method is sensitive to noise and leads to excessive segmentation. Ref. [29] introduces the ant colony algorithm and clustering algorithm. First, the results are obtained through the watershed segmentation method, and then the guidance function in the ant colony algorithm is improved by using the grayscale information and spatial changes after the watershed algorithm is run, thus enabling the ant colony to cluster and merge faster in each area. The limitation is that the watershed point cannot be removed, and the running time of the algorithm after adding the ant colony algorithm is longer than other swarm intelligence algorithms. The traditional maximum entropy segmentation method is sensitive to noise, so it is extended from one-dimensional to two-dimensional to improve the robustness of the algorithm, but two-dimensional is very computationally intensive, and the real-time performance of infrared image segmentation is poor. In view of the above problems, the literature [30] replaces the search method of the original two-dimensional maximum entropy segmentation method with the ant colony algorithm search to reduce the segmentation time and ensure real-time performance. Ref. [31] proposed introducing the ant colony algorithm into the density peak clustering algorithm to find the optimal cluster center and cutoff distance and applied it to medical image segmentation. Second, with the introduction of the ant colony algorithm, optimizing the drawbacks of the ant colony algorithm is also an important way to improve the speed and quality of image segmentation. Ref. [32] combines the original threshold segmentation method with the ant colony algorithm and improves the segmentation accuracy by updating the ant pheromone concentration, improving the initial cluster center, and setting algorithm parameters, thereby reducing the segmentation time. Third, the ant colony algorithm combined with other algorithms to optimize image segmentation is also an effective solution. Ref. [33] introduced a composite ACO k-means segmentation algorithm that took advantage of the advantages of the k-means algorithm and the ant colony algorithm and proposed a new segmentation algorithm. First, set the number of clusters and initialize their centers, then, according to the k-means clustering algorithm, determine that each image pixel belongs to a specific cluster. Ref. [34] combined GEOBIA, F1-score, Taguchi statistical technology, and ACO to improve the detection and mapping of the original date palm. An ant colony algorithm is used to select the most important features. Finally, according to the selected features, the jujube tree was extracted with the help of a decision tree algorithm using a rule-based classification method. In [35], the ant colony clustering algorithm and the improved Markov random field are used together to improve the speed and quality of image segmentation. The ant colony algorithm is used to determine the number of cluster centers, and the FCM algorithm improves the running speed. The algorithm is then improved using an improved Markov random field model to improve its anti-noise ability. In [36], the authors used the ant colony algorithm to strengthen the pulse-coupled neural network when segmenting brain MRI images.

(2) Image matching

Image matching is a fundamental problem in pattern recognition and computer vision and is a prerequisite and key part of image processing tasks. The process of image matching involves establishing the corresponding relationship between feature points. It is disturbed by noise, outliers, and changes in perspective. It is a challenging research topic in image processing. In the application of the ant colony algorithm in the field of image matching, the improvement can be divided into three categories.

In the field of image matching, to overcome the problem that the traditional optimization method for finding stagnation points is easy to fall into the local optimal solution, the ant colony algorithm is introduced. To overcome the shortcomings of the image matching method based on the graph structure model, the literature [37] uses the ant colony algorithm to optimize the objective function and proposes a higher-order graph matching method based on the ant colony algorithm. The algorithm uses the tensor value to calculate the heuristic factor to provide prior knowledge, then calculates the transition probability according to the heuristic factor and the pheromone, and finally uses the searched solution to update the pheromone locally and globally. To overcome the traditional shortcomings, the literature [38] uses the ACO algorithm to optimize the objective function and proposes a higher-order graph matching algorithm based on ACO. In ref. [39], the SIFT feature matching algorithm has many mismatches, and the RANSAC algorithm has many iterations, resulting in slow image stitching and poor effects. The SIFT feature matching algorithm has been improved. That is, the fast-converging ant colony optimization algorithm (FCACO) algorithm is used to optimize the matching point pairs. Third, the ant colony algorithm combined with other algorithms to optimize image matching is also an effective solution. Aiming at the shortcomings of the ant colony algorithm and the characteristics of the artificial fish swarm algorithm, the literature [40] effectively combines the two algorithms, proposes an improved ant colony algorithm, and applies it to image matching. Ref. [41] proposes to divide the features of edge contour images into intersection points, branch points, and general points based on the preprocessing of infrared images with image enhancement and thinning techniques. The calculation method of assigning different weights to these three types of point sets, using ant colony intelligence to optimize the Hausdorff distance, improves the robustness and computational performance of the infrared image matching algorithm.

(3) Image classification

Image classification is the process of simplifying complex phenomena into a small number of general categories and is an important way to extract useful information to achieve target recognition. In today’s massive increase in data volume, how to effectively extract the target or other interesting parts of the image from the background to improve the efficiency and effectiveness of image classification is particularly important.

Given the large number of hyperspectral image bands, which are prone to dimensional disasters, the initial heuristic information provided by the genetic algorithm and the advantages of the optimization capability of the ant colony algorithm are combined to filter and classify the hyperspectral images. The literature [42] proposes a fusion of artificial fish swarm and ant colony algorithms to select the band of hyperspectral images, and the classification has high classification accuracy and efficiency. Ref. [43] proposed a hyperspectral image classification algorithm based on the combination of space-spectral two-dimensional feature ant colony optimization and support vector machine. Ref. [43] proposed a hyperspectral image classification algorithm based on the combination of space-spectral two-dimensional feature ant colony optimization support vector machine. Ref. [44] proposed the fitness function of ant colony according to the Markov property of digital images and established a model of ant colony algorithm to optimize the classification results of remote sensing images based on the coarse classification results of K-means clustering to realize its accurate classification. To improve the accuracy and efficiency of real-time classification of remote sensing images, the literature [45] proposes a real-time classification algorithm for remote sensing image sets based on an ant colony optimization algorithm and an independent feature set. The literature [46] proposes an improved algorithm for texture classification based on genetics. A combination of an ant colony algorithm and an extreme learning machine is proposed, and the random parameters in the network are tried to be ants, and the path of the ants’ foraging process can be regarded as a parameter optimization process. According to the pheromone on each path, the next path can be obtained, and by continuously updating the pheromone, the shortest path can finally be obtained; that is, the parameter optimization is achieved.

(4) Image feature extraction

The key link in image recognition processing—image feature extraction—directly affects the accuracy and speed of image recognition.

In view of the shortcomings of traditional image feature extraction methods, the basic ant colony algorithm is introduced. Ref. [47] proposed an ant colony optimization feature selection algorithm based on information entropy to select the features that separate various samples from each other as far as possible. Ref. [48] proposed a recognition algorithm based on the combination of ant colony optimization and an artificial neural network. This method can prevent the BP network from falling into local minima and has a fast convergence rate. For aircraft image target recognition, the third-order correlation feature, the invariant moment feature, and the Fourier descriptor feature of the image boundary are extracted, and the feature vector is formed as the input vector of the neural network. Third, using other feature extraction methods such as in [49], in order to improve the accuracy and efficiency of weed identification, overlapping leaves were separated by morphological operations and threshold segmentation methods based on distance transformation, and feature selection and classification recognition were performed using ant colony optimization algorithms and support vector machine classifiers to select the optimal features favorable for classification and achieve an optimal combination of features. In ref. [50], given the characteristics of the low resolution and complex background of aerial images, a method of building recognition from aerial images based on the ant colony algorithm and line segment analysis was studied. The ant colony algorithm is applied to the edge extraction of aerial images for the first time, which can effectively remove interference while extracting the target.

(5) Image edge detection

When the traditional ant colony algorithm is applied to image edge detection, there will be problems such as the edge not being smooth enough, being greatly affected by noise, and being easy to converge to the local area. To improve the effect of edge detection, the traditional ant colony algorithm is applied to image edge detection. The literature [51] combined the fast global search ability of genetic algorithms with the global convergence ability of ant colony algorithms and proposed an image edge detection research algorithm based on the genetic ant colony hybrid algorithm. It can improve the quality of image edge detection and greatly shorten the detection time. Ref. [52] proposes an edge detection algorithm based on the ant colony algorithm for UAV image target edge detection because of the environmental specificity of the photos of ships taken by maritime UAVs and the characteristics of image blur caused by UAVs and sea surface shaking. The pheromone left by the artificial ants in the image is displayed in the form of a matrix, and then the optimal path determined by the ant colony is found by the number of pheromones to carry out the edge detection of the image.

The second is the optimization based on the ant colony algorithm itself. Ref. [53] proposed a novel DNA-ant colony algorithm with Levy flight characteristics. The algorithm uses the disturbance of Levy flight characteristics to prevent the basic algorithm from falling into the local optimum and uses DNA crossover and mutation operations to control the algorithm parameters, thereby shortening the search time and improving the search accuracy. Ref. [54] combined the gray gradient with the regional gray mean method to determine the initial position and heuristic matrix of the ants. A weight factor is introduced to define a new probability transfer function, and the pheromone matrix is updated by a chaotic algorithm and adaptive parameters to avoid falling into the local optimum prematurely. The improved algorithm can effectively reduce the influence of noise on edge detection and obtain more complete and clear image edges. The ant colony algorithm, combined with other algorithms, is applied to edge detection. For example, the literature [55] proposes the idea of the bee colony algorithm, which is applied to the problem of image edge detection. At the same time, the maximum inter-class variance strategy is adopted when selecting edges, and the obtained edge information has better integrity.

### 3.4. PSO

#### 3.4.1. Principle of PSO

PSO is inspired by the study of the foraging behavior of birds in the biological world. Kennedy et al. proposed a particle swarm algorithm by studying the behavior of bird flocks as they search for food. In PSO, the maximum food source is defined as the final solution, and a single bird is regarded as an inanimate particle. The search process is as follows:

Initially, random particles are in the space of the final solution, and a single particle is searching for the final solution in the space every moment. Additionally, memorize the closest distance to the final solution during the search process as the current individual extreme value, and then share the distance information with other particles in the particle swarm. The optimal individual extremum among all particle swarms is the global extremum of the particle swarm. Secondly, adjust the direction and speed of the particles through their own individual extreme values and global extreme values. Finally, after several iterations, most of the particles will gather near the final solution.

The inertia factor is defined as ω, *C*_1_ and *C*_2_ are learning factors, also known as acceleration constants, $X_{id}$ is the position of the *i*-th particle in the *d*-dimensional solution space, $P_{gd}$ is called the global extremum, $P_{id}$ is called the individual extremum; the position update formula of the particle is shown in Formula (4); and the velocity update formula is shown in Formula (5):

(4) $$V_{id}=\omega V_{id}+C_{1}random(0,1)(P_{id}-X_{id})+C_{2}random(0,1)(P_{gd}-X_{id})$$

(5) $$X_{id}=X_{id}+V_{id}$$

#### 3.4.2. PSO Algorithm Improvement and Research Status

Compared with the genetic algorithm in the same period, the particle swarm algorithm has the advantage that it does not require many parameters to adjust the search process, and the implementation method is simple. Therefore, it has always been the mainstay of the swarm intelligence algorithm, playing an important role in various industries and fields. Since the official proposal of PSO, various scholars have designed an endless stream of optimization and improvement strategies. These improvements fall into two main categories: improvements to the algorithm itself and the fusion of other algorithms.

The improvement of the algorithm itself, such as improving the parameters in the algorithm, controlling the diversity of the particles in the particle swarm, designing the topological structure of the particles in the initialization and search processes, etc. There are many classic improved algorithms, and the literature [56] puts forward its own suggestions for the setting of parameters in PSO. Firstly, the selection methods of population size, iterative degree, and particle velocity in the algorithm are systematically analyzed. Then, in order to verify the actual influence of these three numbers on the performance of the algorithm, statistical experiments are used to verify the constrained optimization problem. To make the convergence speed in the algorithm more efficient, Shi et al. [57] proposed the concept of inertia weight. The inertia weight is a proportional coefficient related to the moving distance in the previous unit time that controls the influence of the advancing distance in the previous unit time on the current speed, generally written as *ω*. The particle velocity update formula in the algorithm is improved by the idea of inertia weight, and better results are obtained. The PSO algorithm optimized by the other algorithm is also one of the commonly used improvement methods. Ref. [58] proposed a particle swarm algorithm based on natural selection by introducing the natural selection mechanism, calculating the fitness value of particles, and using the selection mechanism to update the particle swarm so as to improve the global optimization ability of particles. Ref. [59] introduced the cuckoo search method to improve the learning efficiency of neural networks for image classification. In recent years, in order to improve the operating efficiency of the algorithm and avoid the PSO from falling into local optimality, the literature [60] has proposed a semi-automatic PSO program. The multimodal function is improved mainly through gradient information and differential control, and the expected results are well obtained through the improvement. Ref. [61] used ant colony and PSO techniques to retrieve the packing peaks of the digital alpha spectrum to solve the second-order packing problem caused by the high count rate in digital alpha spectroscopy.

#### 3.4.3. Improvement and Application of PSO in Image Processing

(1) Image segmentation

The application of the particle swarm algorithm in image segmentation is roughly divided into three types:

First, the particle swarm algorithm was introduced in the direction of the original image segmentation technology. The literature [62] introduced the particle swarm algorithm based on the optimal entropy threshold segmentation technology. The threshold is calculated and selected by the particle swarm algorithm, and the experiment with synthetic aperture radar (SAR) image segmentation shows that this method can reduce the segmentation time while ensuring segmentation accuracy. In [63], the authors improved the defects of watershed image segmentation by particle swarm algorithm and fused particle swarm algorithm and region growing method with it. In [64], the particle swarm algorithm is used to solve the problem that the fuzzy C-means algorithm makes it easy to fall into the local optimum for the segmentation of noisy images. The image is mainly converted into a neuromorphic image, and then a new algorithm combining the particle swarm algorithm and fuzzy C-means algorithm is used to segment the neuromorphic image. Second, because of the problem that the particle swarm algorithm is easy to fall into the local optimum and is slow in the later stages, it proposes an improvement method. To improve the problem that the search ability of particle swarm decreases due to the reduction of population diversity in the later stage of search, literature [65] combines information entropy with the particle swarm algorithm and converts the maximum value of information entropy into a fitness function. Third, optimizing image segmentation by combining PSO and other algorithms is one of the most effective methods at present. In the recent literature, Zhang et al. [66] augmented the ensemble deep neural network with PSO to diversify the search process when performing optic disc (OD) segmentation for retinal images. Liang et al. [67] introduced the particle swarm algorithm into the fuzzy C-means algorithm for the complex situation of segmenting rock images in the process of oil extraction. The binary initial segmentation of the image is realized, and the improved supervised fuzzy C-means algorithm based on the objective function is realized. Farshi et al. [68] improved the color image segmentation technique and optimized the image segmentation algorithm using a multimodal particle swarm. In addition, in the field of deep space exploration, image segmentation for fast orbit detection is a top priority. Ref. [69] combines the PSO algorithm with the gray wolf algorithm to perform multi-level threshold image segmentation. Although the above fusion algorithm further solves the problems of falling into local optimums and low convergence accuracy, the addition of other algorithms inevitably increases the parameters of the improved algorithm, which leads to higher algorithm complexity and a longer running time.

(2) Image matching

With the continuous development of image processing technology, image matching refers to retrieving similar or identical images in an image database based on their characteristics. The traditional image retrieval algorithm requires a large amount of calculation and a small amount of precision. To reduce the amount of calculation and improve accuracy, various scholars proposed introducing the particle swarm algorithm and applying it to image matching. The application of the particle swarm algorithm in image matching is roughly divided into three types:

First, in the direction of introducing the particle swarm algorithm combined with the original image matching technology, Ref. [70] used the grayscale image matching method to analyze the cross marks on the molten iron tanker and to accurately locate the molten iron tanker. In the process of gray-scale image matching analysis, the particle swarm algorithm is used to roughly locate the optimal matching point of the image, and then the improved Harris corner detection algorithm and sub-pixel method are used for precise positioning. In Ref. [71], a fast image matching algorithm based on gray theory and PSO, the GPSO algorithm, is proposed to solve the problems of slow speed and poor noise resistance in image matching. The literature [72] improves the traditional image matching algorithm and the PCA-based image matching algorithm with high mismatch. First, use SIFT to generate a 128-dimensional descriptor vector matrix, and PCA reduces the dimension of the matrix. Then, taking each feature point descriptor vector of one graph as a benchmark, the PSO algorithm is used in the feature point descriptor sub-matrix of another graph to find the global optimal solution of the feature points of the benchmark graph, that is, matching pairs. Second, in view of the problems that the particle swarm algorithm has with falling into local optimums and being slow in later stages, it proposes an improvement method. In [73], to further improve the matching accuracy and computing efficiency of multi-source remote sensing images, a remote sensing image matching algorithm using contourlet transform, Hausdorff distance, and improved particle swarm is proposed. A simplified PSO algorithm with extreme value perturbation is used to match the low-frequency edge images, and the coarse matching points are obtained. Then, according to the position of the coarse matching point, the original image is inverted and calculated, the precise matching is carried out, and finally, the matching of the remote sensing image at full resolution is realized. Third, optimizing image matching by combining PSO and other algorithms is one of the most effective methods at present. Ref. [74] combined particle swarm algorithms and fuzzy neural networks for license plate image matching in intelligent transportation. Utilize the rapidity of PSO and the accuracy of fuzzy neural networks to optimize neural network weight learning and train neural networks.

(3) Image classification

The application of the particle swarm algorithm in image classification can be roughly divided into three types:

First, in the direction of introducing particle swarm algorithm combined with the original image classification technology, Ref. [75] proposed a remote sensing image classification algorithm based on the quantum particle swarm algorithm to improve the classification effect of remote sensing images. The quantum particle swarm algorithm is used to screen the original features of various types of remote sensing images to extract the features that are more important to the classification results of remote sensing images. Finally, a least squares support vector machine is used to establish a remote sensing image classifier to realize remote sensing image classification and recognition. Ref. [76] proposed a method to optimize the FCM cluster center by using the PSO algorithm, which effectively avoided the influence of the traditional FCM due to the initial value and noise, and at the same time, the effect of image segmentation was improved, and the performance was more stable than the traditional FCM method. Second, because of the problems that the particle swarm algorithm has with falling into local optimums and being slow in later stages, it proposes its own improvement method. Ref. [77] proposed a genetic particle optimization algorithm for the classification of images. The selection of the cluster center of the traditional K-Means clustering algorithm has a great influence, and the genetic particle optimization algorithm is introduced to optimize the cluster centers to avoid the random selection of the cluster centers having a great impact on the image classification accuracy.

(4) Image feature extraction

The application of the PSO algorithm in image feature extraction can be roughly divided into three types [78,79]:

First, in the direction of introducing PSO combined with the original image feature extraction technology, the literature [80] proposed an ICA image feature extraction algorithm based on PSO. This algorithm introduces the PSO algorithm into the feature extraction process of the traditional ICA algorithm, which is used to optimize the objective function and can quickly find the global optimal solution of the objective function. Moreover, it can effectively extract image features while greatly reducing the computational complexity of the traditional ICA algorithm. Second, given the problem that the particle swarm algorithm is easy to fall into local optimum and slow in the later stage, it proposes its own improvement method. Ref. [81] proposed a feature extraction algorithm for hyperspectral images based on multi-particle swarm co-evolution. Aiming at the band coding of particles in particle swarms, an improved PSO algorithm, IPSO, is proposed. The update strategy of discrete PSO is used for the band-coding part of particles. Ref. [82] adopts the green pepper target recognition method based on the improved particle swarm algorithm and the least squares support vector machine. The extracted features are divided into training samples and test samples; a mutation strategy is introduced to improve the activity of particle swarms; the optimal parameters of the least squares support vector machine are found; and the recognition model of green pepper training is improved. Third, optimizing image feature extraction by combining PSO and other algorithms is one of the most effective methods at present. In the literature [83], aiming at the problem of fault feature parameter extraction and parameter configuration in the batch diagnosis of power equipment infrared images, the combination of the particle swarm algorithm and the Niblack algorithm is used to segment the equipment thermal image from the background. The parameters, such as the minimum, maximum, and average temperature of the equipment, are extracted, and the sample feature space of the support vector machine is constructed by calculating the temperature rise characteristics of the equipment. In order to reduce the correlation between features and improve the classification speed, the literature [84] used an RBF neural network, a support vector machine, and an Adaboost combined binary particle swarm to adaptively select these tobacco leaf features.

(5) Image edge detection

The application of the particle swarm algorithm in image edge detection is roughly divided into three types:

First, in the direction of introducing PSO combined with the original image edge detection technology, the literature [85] proposes an image edge detection algorithm based on quantum behavioral PSO (QPSO). Second, in view of the problems that the particle swarm algorithm has with falling into local optimums and being slow in later stages, it proposes its own improvement method. Aiming at the problem of the unsatisfactory detection effect of traditional image edge detection methods, literature [86] introduced an improved PSO algorithm into quaternion image edge detection by using the vector rotation principle of quaternion, and a new color image edge detection method is proposed. This method has a better effect on edge detection of color images, can extract image texture details, is stable and easy to converge, and the edge detection speed is also fast. Ref. [87] proposed an edge detection method based on cellular neural networks and the PSO algorithm, aiming at the problem of unsatisfactory edge detail detection in existing gray image edge detection algorithms. Third, optimizing image edge detection by combining PSO and other algorithms is one of the most effective methods at present. The literature [88] proposes an improved PSO algorithm based on the idea of layered difference to solve the problem of premature convergence. The algorithm is introduced into the image edge detection, and the optimal edge of the image is obtained by optimizing the gradient operator. This method can well solve the problem of loss of detail edges and, at the same time, obtain edges in a specified direction. Ref. [89] designed edge detection based on the gray wolf optimization algorithm, and adaptively adjusted the edge detection threshold according to the size of the secret information; the PSO algorithm is used to optimize the embedding bits of edge and non-edge pixels; and the XOR coding is used to embed the secret information after chaotic encryption.

### 3.5. Sparrow Search Algorithm

#### 3.5.1. Principle of Sparrow Search Algorithm

The main idea of the sparrow search algorithm (SSA) is to perform local and global searches by imitating the foraging and anti-predation behaviors of sparrows. The foraging process of sparrows is an algorithm optimization process. The SSA algorithm consists of three types of sparrows: finder, joiner, and scout.

In the SSA algorithm, the solution to the optimization problem is obtained by simulating the foraging process of sparrows. Suppose there are *N* sparrows in a *D*-dimensional search space, then the position of the ith sparrow in the *D*-dimensional search space is $X_{i}=[x_{i1},\cdots x_{id},\cdots x_{iD}],i=1,2,\cdots ,N$, $x_{id}$ represent the position of the *i-*th sparrow in the *d*-th dimension.

The discoverers generally account for 10% to 20% of the population, and the position update formula is:

(6) $$x_{id}^{t+1}=\{\begin{array}{ll} x_{id}^{t}\cdot \exp(\frac{-i}{\alpha T}) & R_{2}<S_{T}\\ x_{id}^{t}+QL & R_{2}\geq S_{T} \end{array}$$

In the formula, *t* is the current iteration number. *T* is the maximum number of iterations. The parameter α is a uniform random number between (0, 1]. *Q* is a random number that obeys the standard normal distribution. The parameter *L* is a matrix of size 1×d with all elements being 1. $R_{2}\in [0,1]$ and $S_{T}\in [0.5,1]$ are the warning value and the safety value, respectively.

Except for the discoverer, the remaining sparrows are used as joiners, and the position is updated according to Formula (7):

(7) $$x_{ib}^{t+1}=\{\begin{array}{ll} Q\cdot \exp\left(\frac{xw_{d}^{t}-x_{id}^{t}}{i^{2}}\right) & i>\frac{n}{2}\\ xb_{d}^{t+1}+\left|x_{id}^{t}-xb_{d}^{t+1}\right|A^{+}\cdot L & \mathrm{Others} \end{array}$$

There is a slight deviation in the original Chinese Formula (7), which is modified as follows:

(8) $$x_{ib}^{t+1}=\{\begin{array}{ll} Q\cdot \exp\left(\frac{xw_{d}^{t}-x_{id}^{t}}{i^{2}}\right) & i>\frac{n}{2}\\ xb_{d}^{t+1}+\frac{1}{D}\sum_{d=1}^{D}\left(rand\{-1,1\}\cdot \left|x_{id}^{t}-xb_{d}^{t+1}\right|\right) & i\leq \frac{n}{2} \end{array}$$

Among them, the parameter *A* is a matrix of dimension 1×D. $xw_{d}^{t}$ is the worst position of the sparrow in the *d*-th dimension at the *t*-th iteration of the population. $xb_{d}^{t+1}$ is the optimal position of the sparrow in the *d*-th dimension at the *t*+1-th iteration of the population.

Sparrows for reconnaissance and early warning generally account for 10% to 20% of the population, and the location updates are as follows:

(9) $$x_{ib}^{t+1}=\{\begin{array}{ll} xb_{d}^{t}+\beta {(x}_{id}^{t}-{\mathrm{xb}}_{d}^{t})\; & f_{i}\neq f_{g}\\ x_{id}^{t}+K(\frac{x_{id}^{t}-xw_{d}^{t}}{\left|f_{i}-f_{w}\right|+e}) & f_{i}=f_{g} \end{array}$$

where parameter β is the step size control parameter, which is a normally distributed random number that obeys the mean value of 0 and the variance of 1. The parameter *K* is a random number between [−1 and 1], which indicates the direction in which the sparrow moves, and is also a step size control parameter. The parameter *e* is a minimal constant to avoid the situation where the denominator is 0; $f_{i}$ is the fitness value of the *i-*th sparrow. The parameters $f_{g}$ and $f_{w}$ are the optimal and worst fitness values of the current sparrow population, respectively.

#### 3.5.2. Algorithm Improvement and Research Status

Although the sparrow search algorithm has been applied in many fields, the main ability of its optimization problem is to rely on mutual cooperation and mutual influence among sparrow individuals. Individuals within the population have no mutation mechanism. After the optimal solution is found, other individuals quickly approach the optimal solution, making it difficult for the algorithm to effectively control the global exploration and local development processes and thus fall into local optimality, resulting in premature convergence of the algorithm. The “premature convergence” of the algorithm is a disadvantage of all swarm intelligence optimization algorithms, and it also exists in the sparrow search algorithm. Based on this defect, scholars have conducted research on its improvement. The major improvement methods can be divided into two categories: improving the algorithm itself and integrating other algorithms.

Improvements to the algorithm itself include changing the method of population initialization, optimizing the update process of individual positions, and introducing mutation operators. There are many classic improved algorithms, and the literature [90] introduced a reverse learning method in the initialization stage of the sparrow population. Ref. [91] introduced the chaotic idea into the population initialization process and adjusted the population through Gaussian mutation and chaotic disturbance to improve the population quality and expand the search range, thereby enhancing the local optimal avoidance ability of the algorithm. Ref. [92] introduced a learning coefficient in the position update part of the searcher and used the mutation operator to improve the position update of the joiner to avoid the algorithm from falling into the local space extreme value. It is also one of the commonly used improvement methods to optimize the sparrow search algorithm by combining it with other algorithms. Ref. [93] improved the position iteration formula of sparrow individuals by combining the flight idea in the bird swarm algorithm to avoid falling into the local optimum due to the rapid assimilation of individuals in the population. Ref. [94] combined the introduction of the butterfly flight method to improve the position update strategy of the discoverer and enhance the global exploration ability of the algorithm. Ref. [95] combined the random following strategy of the chicken swarm algorithm to optimize the position update process of the joiners in the sparrow search algorithm, balancing the local development performance and global search ability of the algorithm.

#### 3.5.3. Improvement and Application of SSA in Image Processing

(1) Image segmentation

The application of the sparrow search algorithm in image segmentation can be roughly divided into three types:

First, after introducing the sparrow search algorithm, optimizing the shortcomings of the sparrow search algorithm is also an important way to improve the speed and quality of image segmentation. Ref. [96] proposes to use the improved sparrow search algorithm to optimize the K-means clustering algorithm for image segmentation. The improved algorithm first introduces the reverse learning strategy of pinhole imaging to update the position of the finder and improve the diversity of the optimal position. Secondly, inspired by the logistic model, a new adaptive factor is proposed to dynamically control the safety threshold to balance the global search and local development capabilities of the algorithm. Ref. [97] proposed a sparrow search algorithm based on the elite reverse learning-Levy flight strategy to address the drawbacks of large calculation amounts, long running times, and inaccurate selection of thresholds, resulting in low segmentation accuracy of the fire image Otsu method segmentation algorithm. To improve the performance of maximum two-dimensional entropy segmentation, the literature [98] proposed a maximum two-dimensional entropy segmentation method based on the improved sparrow search algorithm, and the segmented image with the optimal threshold was obtained by finding the maximum two-dimensional entropy of the image with the improved sparrow search algorithm. Second, the sparrow search algorithm combined with other algorithms to optimize image segmentation is also an effective solution. Ref. [99] proposed a multi-threshold image segmentation method based on the improved sparrow search algorithm to solve the problems of low segmentation accuracy, a large amount of computation, and slow segmentation in traditional multi-threshold image segmentation methods. Combined with the idea of flight behavior in the bird flock algorithm, the sparrow search algorithm is optimized, and then multi-threshold image segmentation based on inter-class variance and Kapur entropy is performed. Ref. [100] used the firefly mechanism to perturb and mutate the optimal solution to further increase the population diversity.

### 3.6. Bat Algorithm

#### 3.6.1. Principle of the Bat Algorithm

The Bat Algorithm (BA) is a new swarm intelligence optimization algorithm based on iterative optimization technology, inspired by bat echolocation predation behavior. Since the algorithm was proposed by Professor Yang in 2010, it has the advantages of a simple model, fast convergence speed, and few parameters [101]. The bats use echolocation technology to detect prey, avoid obstacles, and find habitat in dark environments. It can send out very loud pulses, listen to the echoes bounced back from surrounding objects, and judge the direction and position of the objects according to the different times and intensities of the echoes to the ears [102,103]. Different pulses can also be emitted according to the characteristics of the target prey or obstacle [104].

In the process of simulating the bat algorithm, it is assumed that the search space of bats is *D*-dimensional, and the update rules for the position $x_{i}^{t}$ and velocity $v_{i}^{t}$ of each bat in each generation are given by Formulas (10)–(12):

(10) $$f_{i}=f_{\min}+(f_{\max}-f_{\min})\times \beta$$

(11) $$v_{i}^{t}=v_{i}^{t-1}+(x_{i}^{t}-X_{*})\times f_{i}$$

(12) $$x_{i}^{t}=x_{i}^{t-1}+v_{i}^{t}$$

where β∈[0,1] is a random vector, $X_{*}$ is the current local optimal solution (position) in the group, $f_{i}$ is the frequency of the sound wave emitted by the bat, and the adjustment interval is $\left[f_{\min},f_{\max}\right]$. During the experiment, the corresponding frequency variation interval can be set according to the needs of the problem [105].

For local search, once a solution is selected among the current best solutions, new local solutions are generated using random walks.

(13) $$x_{new}=x_{old}+\varepsilon \times A^{t}$$

where ε∈[−1,1] is a random number, and *A* is the average loudness of the entire population in the same generation.

The acoustic loudness $A_{i}^{t+1}$ and frequency $r_{i}^{t+1}$ of the *i*-th bat are updated using Equations (14) and (15).

(14) $$A_{i}^{t+1}=\alpha \times A_{i}^{t}$$

(15) $$r_{i}^{t+1}=r_{i}^{0}[1-\exp(-\gamma \times t)]$$

Among them, α∈(0,1) is the acoustic loudness attenuation coefficient; γ>0 is the pulse frequency enhancement coefficient; $r_{i}^{0}$ is the initial pulse frequency of bat *i*.

For arbitrary α and γ, when t→∞, there are $A_{i}^{t}\to 0$, $r_{i}^{t}\to r_{i}^{0}$. When $A_{i}^{t}$ tends to 0, it can be considered that the bat finds the prey and temporarily does not send out the pulse, and the variation range of the pulse can be set to different value intervals according to the needs of the problem [106].

#### 3.6.2. Improvement of the Bat Algorithm and Research Status

Although the bat algorithm has been applied in many fields, the main ability of its optimization problem is to rely on mutual cooperation and mutual influence between bat individuals. Individuals within the population have no mutation mechanism. Once the local optimal value is searched, it will fall into place and influence other individuals to move closer to it, causing the algorithm to converge prematurely and greatly reducing the diversity of the population [107]. The “premature convergence” of the algorithm is a disadvantage of all swarm intelligence optimization algorithms, and it also exists in the bat algorithm. Based on this defect, many scholars have proposed a variety of strategies to improve the algorithm, thereby improving its performance.

The improvement of the algorithm itself, such as improving the parameters in the algorithm, controlling the diversity of the population, etc. There are many classic and improved algorithms. Guo et al. [108] used different chaotic mapping strategies to improve the bat algorithm and proposed the chaotic bat algorithm, which replaced the constant parameters in the basic algorithm with chaotic maps. Lin et al. [109] also proposed using a linear decreasing (increasing) function to multiply the chaotic mapping function to calculate the loudness of the pulse and compared the influence of different mapping functions on the performance of the algorithm. Optimizing the bat algorithm by combining other algorithms is also one of the commonly used improvement methods.

#### 3.6.3. Improvement and Application of BA in Image Processing

(1) Image segmentation

The application of the bat algorithm in image segmentation can be roughly divided into three types:

First, the bat algorithm is introduced and combined with the original image segmentation technology. Ref. [110] designed a kernel fuzzy C-means algorithm based on bat algorithm optimization to segment hard exudates. Using the kernel idea, the low-dimensional operation space of the traditional fuzzy C-means algorithm is converted to high-dimensional, thereby improving its clustering performance, and the initial clustering center is optimized by the bat algorithm to improve the segmentation accuracy of hard exudates. Ref. [111] proposed a maximum entropy engineering drawing segmentation algorithm based on the bat algorithm. By introducing a binary coding mechanism to simulate the coding, translation, and expression of genes in genetics, the initialization of the bat position in the BA algorithm is optimized to make it easy to constrain the initial space of the position and generate a well-diversified population. Ye et al. [112] took image thresholding as a constrained optimization problem and obtained the optimal threshold through the convergence of the BA algorithm. The bat algorithm is used to optimize the two-dimensional information entropy, aiming at the difficult and time-consuming problem of threshold selection in the process of thermal infrared image segmentation of power equipment. In the literature [113], aiming at the difficulty of threshold selection and time-consuming problem in the process of thermal infrared image segmentation of power equipment, the bat algorithm is used to optimize the two-dimensional information entropy. By analyzing the two-dimensional information entropy threshold segmentation principle, the bat algorithm is introduced to quickly search for the optimal segmentation threshold, and threshold segmentation experiments are carried out on infrared images of several substation equipment. Second, after the introduction of the bat algorithm, the shortcomings of poor self-adaptability and low search accuracy of the optimized bat algorithm are also important ways to improve the speed and quality of image segmentation. Ref. [114] proposed a bat-optimized two-dimensional Tsallis entropy multi-threshold SAR image segmentation algorithm, using cubic mapping to homogenize the initial bat population and introducing Levy flight features to enhance the algorithm’s ability to jump out of local optimality. Ref. [115] studied the image segmentation algorithm of the BP neural network optimized by the bat algorithm. The initial weights and thresholds of the BP neural network were optimized by the bat algorithm, and the gray-value image of a surgical instrument marker was used as a sample to train the neural network and then image segmentation. The image segmentation effect is clearer than the ordinary method. Alihodzic et al. [116] introduced the crossover factor of differential evolution and artificial bee colonies based on the original algorithm to search for the best threshold. Scholz et al. [117] proposed a stochastic bounce algorithm that combines the bat algorithm with target tracking.

(2) Image classification

By improving the bat algorithm, the effectiveness of image classification is enhanced. Ref. [118] used the binary bat algorithm to solve classification and feature selection problems. Ref. [119] used an optimized binary bat algorithm to classify leukocytes. Mainly extract some features from WBC images, and then use optimization algorithms to obtain necessary feature subsets from high-dimensional data to classify different types of white blood cells. In blood analysis, the optimized bat algorithm has fast classification and high accuracy, which helps doctors diagnose diseases.

(3) Image edge detection

Ref. [120] first uses the improved bat algorithm to optimize the Otsu method to obtain the best threshold. A binary image is obtained by segmenting the image with the best threshold. Then the Canny operator is used to detect the edge of the binary image to extract the edge information of the defect. Among them, the low threshold of Canny edge detection is the lowest gray level of the preprocessed image, and high threshold is the best in the threshold segmentation.

### 3.7. The Salp Swarm Algorithm

#### 3.7.1. The Principle of Salp Group Algorithm

The Salp Swarm Algorithm (SSA) is a global optimization algorithm based on swarm intelligence proposed by Mirjalili et al. in 2017 [121]. The salp is a free-floating tunican, a marine creature whose body organization and movement are highly similar to those of jellyfish. In the chain-like group behavior of salps, individuals usually connect end to end to form a “chain” that moves in sequence. In the salps chain, divided into leaders and followers, the leader moves towards the food and directs the movement of the followers that follow; followers move according to a strict “hierarchy” system, only affected by the previous salp. Such a movement mode enables the salps chain to have strong global exploration and local development capabilities.

Population initialization: Let the search space be the Euclidean space of D×N, *D* is the space dimension, and *N* is the number of populations. The location of salps in space is represented by $X_{n}={\left[X_{n1},\;X_{n2},\;X_{n3},\;\cdots ,\;X_{nD}\right]}^{T}$, and the location of food is represented by $F_{n}={\left[F_{n1},\;F_{n2},\;F_{n3},\;\cdots ,\;F_{nD}\right]}^{T}$, n=1, 2, 3, ⋯, N. The upper bound of the search space is $ub=[ub_{1},\;ub_{2},\;ub_{3},\;\cdots ,\;ub_{j},\;\cdots ,\;ub_{D}]$, and the lower bound is $lb=[lb_{1},\;lb_{2},\;lb_{3},\;\cdots ,\;lb_{j},\;\cdots ,\;lb_{D}]$, j=1, 2, 3, ⋯, N.

(16) $$X_{D\times N}=rand(D,N)\cdot (ub-lb)+lb$$

In the population, the leader is represented by $X_{d}^{l}$, and the followers are represented by $X_{d}^{i}$, i=1, 2, 3.4, ⋯N;d=1, 2, 3, ⋯D.

Leader position update: During salp chain movement and foraging, the leader’s position update is expressed as:

(17) $$x_{d}^{l}=\{\begin{array}{l} F_{d}+c_{1}((ub-lb)c_{2}+lb),c_{3}\geq 0.5\\ F_{d}-c_{1}((ub-lb)c_{2}+lb),c_{3}<0.5 \end{array}$$

where $X_{d}^{l}$ and $F_{d}$ are the position of the first salps (leader) and food in the d-th dimension, respectively, the parameters *ub* and *lb* are the corresponding upper and lower bounds, respectively. Among them, $c_{1}$, $c_{2}$, $c_{3}$ are control parameters.

Equation (17) shows that the leader’s location update is only related to the location of the food. $c_{1}$ is the convergence factor in the optimization algorithm, which plays the role of balancing global exploration and local development and is the most important control parameter in SSA. The expression for $c_{1}$ is:

(18) $$c_{1}=2e^{-{\left(\frac{4l}{L}\right)}^{2}}$$

where *l* is the current iteration number and *L* is the maximum iteration number. The convergence factor is a decreasing function from 2 to 0.

The control parameters $c_{2}$ and $c_{3}$ are random numbers of [0,1], which are used to enhance the randomness of $X_{d}^{l}$ and improve the global search and individual diversity of the chain group.

Follower position update: During the movement and foraging process of the salp chain, the followers move forward in a chain-like manner through the mutual influence between the front and rear individuals. Their displacement conforms to Newton’s law of motion, and the follower’s motion displacement is:

(19) $$X=\frac{1}{2}at^{2}+v_{0}t$$

where *t* is the time; *a* is the acceleration, calculated as $a=(v_{final}-v_{0})/t$; $v_{0}$ is the initial velocity, and $v_{final}=(X_{d}^{i}-X_{d}^{i-1})/t$.

Considering that in the optimization algorithm, *t* is iterative, set *t* = 1 in the iterative process, and $v_{0}=0$. Then Formula (19) can be expressed as:

(20) $$X=\frac{X_{d}^{i}-X_{d}^{i-1}}{2}$$

where i≥2, $X_{d}^{i}$ and $X_{d}^{i-1}$ are the positions of two salps that are closely connected to each other in the d-dimension, respectively. Therefore, the position of the follower is expressed as:

(21) $$X_{d}^{i\prime }=\frac{X_{d}^{i}+X_{d}^{i-1}}{2}$$

where $X_{d}^{i\prime }$ and $X_{d}^{i}$ are the updated follower’s position and the pre-update follower’s position in the *d*-th dimension, respectively.

#### 3.7.2. Algorithm Improvement and Research Status

Although the salps swarm algorithm has been applied in many fields, it has some shortcomings. For example, it is easy to fall into the local optimal solution in the iterative process, and it is easy to appear premature when solving high-dimensional problems, and the convergence speed and convergence accuracy of the algorithm are not high. Based on this shortcoming, many scholars have proposed a variety of strategies to improve the Salp Swarm Algorithm’s performance. These improvements fall into two main categories: improvements to the algorithm itself and the fusion of other algorithms.

The improvement of the algorithm itself, such as improving the parameters in the algorithm, generating the initial population from the chaotic map, updating the position, etc. There are many classic improved algorithms, and the update of the leader in the literature [122] introduces the elite individual hybrid mutation strategy. Adopt an adaptive differential mutation strategy for follower updates to speed up optimization efficiency. Ref. [123] discretizes and improves the leader’s position update method based on Lévy flight; adaptive inertia weight is introduced into the follower’s position update formula. To improve search efficiency, a crossover operator and a mutation operator based on the critical path are designed to ensure the diversity of the population. Ref. [124] added inertia weight to the leader position update formula, and introduced a leader-follower number adaptive adjustment strategy in the selection of global and local searches. Ref. [125] designs a leader update mechanism based on logistic mapping to effectively enhance population diversity. Use the follower update mechanism based on dynamic learning to improve global search ability. Design an adaptive adjustment mechanism for the leader/follower scale to effectively balance the local development and global exploration capabilities of the population. It is also one of the commonly used improvement methods to optimize the Salps Swarm Algorithm by combining it with other algorithms. Ref. [126] added elite reverse learning and differential strategy based on the Salps Swarm Algorithm to identify the parameters of the two-diode model of photovoltaic cells. Ref. [127] introduced the cuckoo search operator into the Salps Swarm Slgorithm to enhance its global search ability, and finally used the improved Salps Swarm Algorithm to optimize the multi-carrier modulator for visible light communication. In [128], to enhance the development and exploration ability of the salps algorithm, Brownian motion was introduced into the salps algorithm, and the optimization performance of the algorithm was improved through the random motion mechanism of particles. Ref. [129] proposed an improved Salps Swarm Algorithm based on normal process search and differential evolution—the double-leader Salps Swarm Algorithm.

#### 3.7.3. Improvement and Application of SSA in Image Processing

(1) Image segmentation

The application of the salps group algorithm in image segmentation can be broadly divided into three types:

First, in the direction of introducing the salp swarm algorithm combined with the original image segmentation technology. In [130], to solve the problem of high space and time complexity in the multi-threshold segmentation of images by the Otsu method, the SSA algorithm is combined with the traditional Otsu segmentation algorithm (SSA-Otsu) to optimize the effect of multi-threshold segmentation of color images. Ref. [131] uses the region-growing method to segment color images, which has certain subjectivity and limitations. Crack grayscale image segmentation methods include the Canny algorithm, iterative methods, maximum inter-class variance methods, particle swarm algorithm, and Salps Swarm Algorithm. The image segmentation algorithm based on salp group optimization proposed in this paper has the advantages of a short time, a small amount of calculation, and a good segmentation effect. Second, after the introduction of the Salps Swarm Algorithm, optimizing its shortcomings is also an important way to improve the speed and quality of image segmentation. In [132], to improve the effect of medical image segmentation, a fractal search mechanism and a Gaussian skeleton mechanism are introduced into the original SSA. The optimal threshold value in multi-threshold image segmentation is searched by non-local mean and Kapur entropy. Third, it is also an effective solution to optimize image segmentation by combining it with other algorithms. Ref. [133] proposed a dual-branch blood vessel segmentation method based on an improved particle swarm-salp algorithm for RGB blood vessel image segmentation. To effectively improve the measurement accuracy, Ref. [134] introduced the Scaramuzza model for fisheye camera calibration and realized the calculation of the parameters of the tree height measurement model. The tree image segmentation was performed by improving the FCM algorithm, and the superpixel algorithm was used for image preprocessing to reduce the computational complexity.

(2) Image matching

In the literature [135], a new image matching method of the Salps Swarm Algorithm is proposed to solve the problems of many parameter adjustments and low matching accuracy in the traditional swarm intelligence optimization image matching algorithm. To solve the problems of slow convergence speed and poor calculation accuracy in the optimization process of the Salps Swarm Algorithm (SSA), a new salps swarm algorithm (NSSA) was proposed in [136]. First, analyze the deficiencies of salps in SSA in the process of following leaders. Then, the idea of following the leader wolf in the gray wolf optimization algorithm is used to improve the way the salps follow the leader, and the algorithm is applied to image matching, which has better convergence speed, calculation accuracy, and robustness.

(3) Image classification

To achieve better classification performance on medical diagnosis problems, the literature [137] introduced the salps algorithm to optimize the initial cluster center of the FCM algorithm to solve the problem that the FCM algorithm is sensitive to the initial cluster center. In this way, the accurate extraction of tree extreme points is realized, and the extreme points are used as the input of the tree height measurement model to realize the tree height measurement.

(4) Image feature extraction

Ref. [138] proposed a feature selection method for converter steelmaking based on the improved Salps Swarm Algorithm. First, the number of selected features is preset before feature selection to reduce the randomness of feature selection; then the original algorithm is used to generate candidate features, while the leader is updated by selecting features from a subset of candidate features using a probability function [139]. Finally, the latter is used to follow the former’s salp group foraging mechanism to update the followers and increase the exploration ability of the optimization algorithm. Using this method, the fitness function value is reduced to the optimal value, the optimal subset of features is selected from the converter steelmaking process data, and irrelevant and redundant features are eliminated, which is necessary for end-point carbon temperature prediction [140,141,142].

## 4. Comprehensive Analysis and Summary of Swarm Intelligence Optimization Algorithm

### 4.1. Comprehensive Analysis and Comparison of Image Segmentation

In the use scenarios of the swarm intelligence algorithm combined with image segmentation technology, most researchers apply it in the field of medical image segmentation [143,144]. For example, it is applied to CT images, MRI images, etc., and some researchers also apply it to infrared images, synthetic aperture radar images, etc. The selection of these application images is more dependent on the applicable rules of the original image segmentation method before the combination algorithm. In Table 1, some examples of the ant colony algorithm, particle swarm algorithm, sparrow search algorithm, bat algorithm, and Salps Swarm Algorithm combined with image segmentation technology are sorted out by combining the references cited above, and the list is analyzed and summarized [145,146,147,148,149].

It can be obtained by analyzing and summarizing the information in the comprehensive table. The swarm intelligence algorithm often combines three image segmentation techniques: first, it is used in combination with the threshold segmentation method, and the optimal threshold is obtained through the algorithm [150,151]. Second, combined with the clustering algorithm, the clustering center and other parameters that need to be set before are automatically obtained by the algorithm. Third, it is used in combination with neural networks or machine learning to optimize the algorithm through its own advantages or to perform preprocessing before recognition and segmentation [152,153].

It can be seen that the swarm intelligence algorithm is an excellent optimization method and tool that can effectively solve some of the defects of the original algorithm and obtain better results. However, the introduction of the swarm intelligence algorithm cannot completely change the inherent drawbacks of the algorithm [154,155]. For example, in threshold segmentation, when the difference between the target and the background grayscale is extremely low, the result is still poor. In addition, in the process of introducing new algorithm improvements, the disadvantages of the new algorithm itself will inevitably be introduced. These issues remain to be resolved [156].

### 4.2. Comprehensive Analysis and Comparison of Image Matching

Table 2 sorts out some examples of swarm intelligence optimization algorithms combined with image matching technology by combining the references cited above and makes a list analysis and summary.

It can be obtained by analyzing and summarizing the information in the comprehensive table. The swarm intelligence algorithm often combines three image matching techniques: First, the feature point matching is performed by combining it with artificially designed detectors, such as the method based on image grayscale, neighborhood pixel detection, and SIFT. Second, it is used in combination with neural networks (such as fuzzy neural networks) or machine learning to optimize the algorithm through its own advantages [157].

In the use scenarios of the swarm intelligence algorithm combined with image segmentation technology, the scholars apply it in the field of medical image matching. For example, it is applied to CT images, brain images, etc. It is also applied to infrared images of power equipment, license plate image matching, remote sensing image matching, etc. [158].

### 4.3. Comprehensive Analysis and Comparison of Image Classification

Table 3 sorts out some examples of swarm intelligence optimization algorithms combined with image classification technology by combining the references cited above and makes a list analysis and summary.

Through the analysis and summary of the information in the comprehensive table, the swarm intelligence algorithm often combines three image segmentation techniques: first, combined with SVM. Second, combined with the methods of FCM cluster center and K-means clustering, pre-processing before classification is carried out. Third, it is used in combination with neural networks or machine learning to optimize the algorithm through its own advantages.

In the use scenarios of the swarm intelligence algorithm combined with image segmentation technology, most researchers apply it in the field of medical image classification, such as medical pathology images, liver B-ultrasound images, WBCs images, white blood cell classification, etc. Some researchers have also applied it to forest remote sensing images, high-precision classification of vegetables and fruits, and moving images.

### 4.4. Comprehensive Analysis and Comparison of Image Feature Extraction

Table 4 sorts out some examples of swarm intelligence optimization algorithms combined with image feature extraction technology by combining the references cited above and performing list analysis and summary.

By analyzing and summarizing the information in the comprehensive table, the swarm intelligence algorithm often combines three image segmentation techniques. First, combined with SIFT (Scale Invariant Feature Transform), find key points (feature points) in different scale spaces and calculate the direction of the key points. Second, combined with HOG (Histogram of Orientation Gradients), features are formed by calculating and counting the gradient direction histograms of local areas of the image. Third, it is used in combination with the neural network to optimize it through the advantages of the algorithm itself or to perform preprocessing before recognition and segmentation.

In the use scenarios of the swarm intelligence algorithm combined with image segmentation technology, most researchers apply it in the field of medical image feature extraction, and some researchers apply it in infrared images of power equipment, weed identification, and so on.

### 4.5. Comprehensive Analysis and Comparison of Image Edge Detection

Table 5 sorts out some examples of swarm intelligence optimization combined with image edge detection technology by combining the references cited above and performing a list analysis and summary.

By analyzing and summarizing the information in the comprehensive table, the swarm intelligence algorithm often combines three image segmentation techniques. First, the introduction of basic edge detection filters such as Sobel, Prewitt, Roberts operator, Canny operator, Marr–Hildreth operator, and so on. Use filters to improve the performance of noise-dependent edge detectors. Second, combine the gray gradient and regional gray mean methods. Third, it is used in combination with the cellular neural network to optimize it through the advantages of the algorithm itself.

In the use scenarios of the swarm intelligence algorithm combined with image edge detection technology, most researchers apply it in the field of medical images, such as CT images, liver images, and so on. Some researchers also apply it to infrared images, color images, texture images, asteroid surface image segmentation, UAV image object edge detection, etc. The selection of these application images is more dependent on the applicable rules of the original image segmentation method before the combination algorithm.

It can be seen that the swarm intelligence algorithm is an excellent optimization method and tool that can effectively solve some of the defects of the original algorithm and obtain better results. However, the introduction of the swarm intelligence algorithm cannot completely change the inherent drawbacks of the algorithm. For example, in threshold segmentation, when the difference between the target and the background grayscale is extremely low, the result is still poor. In addition, in the process of introducing new algorithm improvements, the disadvantages of the new algorithm itself will inevitably be introduced. These issues remain to be resolved.

## 5. Conclusions and Future Work

### 5.1. Conclusions

Applying a bionic intelligent optimization algorithm to solve complex image processing problems has a good development prospect. In this paper, the development process of the swarm intelligence algorithm is systematically discussed, and a large number of documents prove the improvement of the algorithm and several typical combination methods and improvement methods applied in the field of image processing. Through the rapid development in recent years, whether it is the classic ant colony algorithm and particle swarm algorithm, the sparrow search algorithm, the bat algorithm, and the salps swarm algorithm, the theory and foundation of swarm intelligence algorithms represented by emerging algorithms in recent years have been gradually improved.

For some complex image processing problems that are difficult to establish a strict mathematical model for, the image processing problem can be transformed into a function optimization problem. The self-organization of the bionic intelligent optimization algorithm is especially suitable for dealing with complex problems that are difficult or even impossible to solve by traditional optimization methods. nonlinear problem. Therefore, the complex digital image processing problem can be transformed into the problem of optimizing and solving the objective function by using the bionic intelligent optimization algorithm. First, determine the objective function of the image processing problem, that is, establish an objective function to measure the quality of the image processing problem, and transform the image processing problem into a problem of solving the maximum or minimum value of the objective function; second, choose a suitable bionic intelligent optimization algorithm. Optimize and solve the objective function. For complex image processing problems, use the bionic intelligent optimization algorithm to optimize and solve the function. The powerful optimization ability of the algorithm can simplify complex problems.

The following issues have always remained, despite the fact that swarm intelligence algorithms are evolving quickly and offer many advantages in tackling engineering challenges involving complicated information. First off, there is not enough mathematical theory, and the concept and foundation of the system are derived from the simulation of natural biological groupings. Second, the swarm intelligence algorithm may encounter the issue of local optimality during the optimization process because it searches for optimization through repeated iterations in the solution space, and the control of the convergence speed in the early and late stages of the algorithm is not precise. Thirdly, there is a lack of uniformity in parameter design standards due to the artificial starting values. When it comes to image segmentation, in addition to the issues mentioned above, there are other issues such as lengthy processing times caused by combining multiple image processing technologies, subpar results obtained when high-precision image processing is required due to the randomness of algorithm results, etc.

### 5.2. Future Work

Swarm intelligence algorithms continue to be a key area of study in the science of artificial intelligence algorithms, and new swarm intelligence algorithms such as the dragonfly, squirrel, and butterfly algorithms will likely be developed in the future. The innovative swarm method should enhance the mathematical formula’s derivation and demonstration, derive the initial parameters’ unified values using rigorous mathematical theory, and avoid relying on empirically discovered beginning values. The swarm intelligence algorithm should continue to be combined with other cutting-edge advanced technologies in order to strengthen the analysis of the algorithm on location update rules. It should also continuously address its own flaws, overcome its own constraints, and enhance the performance of other advanced technologies.

## Figures and Tables

**Table 1:** Image segmentation comprehensive analysis.

	Algorithm	Briefly Analyze	Image Segmentation	Advantage	Disadvantage	Applicable Field
ACO	[29]	Improving Watershed Segmentation	watershed segmentation	Improved over-segmentation	The watershed point still exists, long segmentation time	Pelvic bone CT images, etc.
	[31]	Improve clustering algorithm	Cluster segmentation method	The cluster center is automatically generated by the algorithm	Further increase the parameters and lengthen the segmentation time	Medical images, brain MRI images
	[32]	Improved ACO algorithm and combined threshold segmentation	Threshold segmentation	Improve the speed of the algorithm and reduce the segmentation time	The clarity has not improved, and the algorithm parameters are still artificially set	Medical CT images
	[33]	Fusion of k-means algorithm and ACO algorithm	k-means clustering algorithm	Improved anti-noise capability, improved segmentation accuracy	The segmentation time is still long	Medical MRI images
	[36]	ACO algorithm enhanced impulse coupled neural network	Pulse coupled neural network	The parameter setting is easy to set	Depends on the dataset, the segmentation time varies little	MRI image of the brain
PSO	[62]	Improved optimal entropy threshold segmentation	Best entropy thresholding segmentation technique	Automatically select threshold	Long segmentation time	Synthetic aperture radar (SAR) map
	[63]	Improved watershed segmentation method	watershed segmentation method, regional growth	Improved the bug of over-segmentation	The segmentation accuracy is still not high, and the time increases	Brain MRI images
	[65]	Combined with information entropy threshold segmentation	Information entropy threshold segmentation	Reduce image segmentati and enhance anti-noise ability	The sharpness improvement rate is not high	Medical CT images
	[66]	Reinforced ensemble deep neural networks	Integrated deep neural network	Improved segmentation accuracy and optimized learning parameters	Depends on the dataset, may become stuck in a local optimum	Retinal image segmentation and diabetic macular edema detection
	[69]	Combined with gray wolf algorithm for threshold segmentation	Threshold segmentation	Improved accuracy for segmenting complex images	Segmenting time varies	Surface image segmentation
SSA	[91]	Combined with Otsu algorithm	Otsu algorithm	Automatic threshold selection, less segmentation time	The segmentation accuracy is not significantly improved	Ship synthetic aperture radar image
	[96]	Optimizing K-means clustering algorithm	K-means clustering algorithm	Improve the diversity of optimization positions	Long segmentation time	Medical image
	[97]	Combined with exponential entropy multi-threshold image segmentation method	Exponential entropy multi-threshold image segmentation	Small amount of calculation	The threshold selection is not accurate enough, resulting in low segmentation accuracy	Forest fire images
	[98]	Combined with maximum two-dimensional entropy segmentation method	Maximum two-dimensional entropy segmentation method	Reduce the amount of calculation and shorten the calculation time	The segmentation accuracy is not high	Medical image
	[93]	Combining bird flock algorithm, Otsu algorithm and Kapur entropy	Otsu’s algorithm and Kapur entropy	High segmentation accuracy, fast segmentation speed	Long segmentation time	Medical image
BA	[110]	Optimized fuzzy c-means algorithm	Fuzzy c-means algorithm	Improved segmentation accuracy for hard exudates	Long segmentation time	Hard exudate image
	[111]	Combined with maximum entropy threshold segmentation	Maximum entropy threshold segmentation	Improved segmentation accuracy	Long segmentation time	Engineering drawing image segmentation
	[113]	Optimizing two-dimensional information entropy threshold segmentation	Two-dimensional information entropy threshold segmentation	Threshold selection is simple and time-consuming	Low definition	Thermal infrared image of power equipment
	[114]	Two dimensional Tsallis entropy multi threshold segmentation method	Two dimensional Tsallis entropy multi threshold segmentation method	Strengthen the ability of the algorithm to jump out of the local optimum	The segmentation accuracy is not high	SAR images
	[115]	Optimizing BP neural network	BP neural network	Clearer segmentation than ordinary methods	Long segmentation time	Grayscale image of surgical instrument markers
SALP	[130]	Combined with Otsu algorithm	Otsu algorithm	Low space and time complexity	Fall into a local optimum	Color image
	[131]	Combined region growing method	Region growing method	Small amount of calculation, good segmentation effect	Small change in segmentation time	Crack image
	[132]	Combining nonlocal mean and Kapur entropy	Nonlocal mean and Kapur entropy	Short time consuming	Easy to fall into local optimum	Medical image
	[133]	Combined with PSO	PSO algorithm	Good segmentation effect	Long segmentation time	RGB vessel image
	[134]	Improved FCM algorithm	FCM algorithm	Improve measurement accuracy	Long segmentation time	Tree images

**Table 2:** Comprehensive analysis of image matching.

	Algorithm	Briefly Analyze	Image Segmentation	Advantage	Disadvantage	Applicable Field
ACO	[37]	Improved high order graph matching algorithm	High order graph matching algorithm	Improved matching efficiency	Easy to fall into local optimal solution	Medical image
	[38]	Improved SIFT feature algorithm	Principal component analysis and kernel projection	Improve the matching efficiency of feature points	The search speed is slow and the matching accuracy is not high	Medical CT images
	[40]	Combined with artificial fish swarm algorithm	Artificial fish swarm algorithm	Improve matching efficiency	Matching accuracy is not high	Brain image
	[39]	Improved SIFT algorithm	SIFT algorithm	Effectively eliminate mismatch points and reduce matching time	Easy to fall into local optimum	Infrared image of power equipment
	[41]	Optimizing Hausdorff distance	Intelligent optimization of Hausdorff distance	Improved robustness	Large amount of calculation	Infrared image matching
PSO	[70]	Combined with grayscale image matching methods	Improved Harris corner detection algorithm and sub-pixel method	Precise positioning	Slow search speed	Cross sign on hot metal tank car
	[71]	Combined with grey theory	Grey theory	Significantly improved matching speed and robustness	Easy to fall into local optimum	Medical image
	[72]	Combining PCA, SIFT algorithm	PCA and SIFT algorithm	Reducing false matching of image matching algorithm	Low matching accuracy	Medical image
	[73]	Combining Contourlet transformation and Hausdorff distance	Contourlet transform, Hausdorff distance	Improve the matching accuracy and computing efficiency	Large amount of calculation	Remote sensing image matching
	[74]	Combined with fuzzy neural network	Fuzzy neural network	High matching efficiency	Easy to fall into local optimum	License plate image matching
SALP	[135]	Combined with chaos theory	Chaos theory	High matching accuracy and efficiency	Long match time	Medical image
	[136]	Combined with gray wolf algorithm	Gray wolf algorithm	Has better convergence speed and calculation accuracy	Easy to fall into local optimum	Medical CT image

**Table 3:** Comprehensive analysis of image classification.

	Algorithm	Briefly Analyze	Image Segmentation	Advantage	Disadvantage	Applicable Field
ACO	[42]	Artificial fish swarm—ACO algorithm fusion	Artificial fish swarm algorithm	Has high classification accuracy and efficiency	Easy to fall into local optimum	Hyperspectral imagery
	[43]	Combinatorial optimization support vector machine classification	Support vector machine	Improve the classification accuracy of SVM algorithm	Classification takes a long time	Hyperspectral imagery
	[44]	Combined with K-means clustering	K-means clustering	Overcome the slow convergence of the algorithm	Long time classification	Forest remote sensing images
	[45]	Real time classification algorithm based on independent feature set	Real time classification algorithm	Improve real-time classification accuracy and efficiency	Low classification accuracy	Remote sensing image set
	[46]	Combined with extreme learning machine	extreme learning machine	Get higher texture classification effect	Easy to fall into local optimum	Texture classification
PSO	[75]	Combined with least squares support vector machine	Least squares support vector machine	Improve the classification effect of images	Low classification accuracy	Remote sensing image
	[76]	Optimizing FCM cluster center method	FCM method	Avoid the influence of initial value and noise	Easy to fall into local optimum	Moving image
	[77]	Optimized support vector machine algorithm	Support vector machine algorithm	Improve the accuracy of image classification	Long time classification	Medical pathology image
	[78]	Optimized mixed kernel ELM model	Mixed kernel ELM model	Real time, high precision	Easy to fall into local optimum	High precision classification of vegetables and fruits
	[79]	Combined with genetic algorithm	Genetic algorithm	High image classification accuracy	Low classification accuracy	Medical image
BA	[118]	Binary bat algorithm	Binary bat algorithm	Can quickly classify	Classification accuracy is not high	Medical image
	[119]	Optimized binary bat algorithm	Binary bat algorithm	Has fast classification and high accuracy	Easy to fall into local optimum	WBCs images, leukocyte classification
	[139]	Combined with FCM clustering algorithm	FCM clustering algorithm	Can quickly classify	Low classification accuracy	Breast cancer images, liver disease images
	[140]	Combined with genetic algorithm	Genetic algorithm	Reduce classification error	Easy to fall into local optimum	Remote sensing image
SALP	[132]	Optimizing the FKNN model	FKNN model	Better convergence accuracy	Long time classification	Medical image
	[134]	Combined with FCM algorithm	FCM algorithm	Accurate extraction of tree extreme points	Long time classification	Image calculation of tree height

**Table 4:** Comprehensive analysis of image feature extraction.

	Algorithm	Briefly Analyze	Image Segmentation	Advantage	Disadvantage	Applicable Field
ACO	[47]	Ant colony optimization feature selection algorithm based on information entropy	Feature selection algorithm	Features that are separated from each other	Large amount of calculation	Medical image
	[43]	Combined with pulse coupled neural network	Pulse coupled neural network	Improve image recognition accuracy	Low feature extraction accuracy	Medical image recognition
	[48]	Combined with artificial neural network	Artificial neural network	Shorten the training time	Easy to fall into local optimization	Medical image
	[49]	Support vector machine classifier	Support vector machine classifier	Improve the accuracy and efficiency	Long time consuming	Weed image recognition
	[50]	Combined with optimal eigenvector selection method	Feature vector selection method	Avoid local convergence and improve the search efficiency	Low accuracy	Aerial building image recognition
PSO	[80]	Introducing the ICA algorithm	ICA algorithm	Reduce the computational complexity of the algorithm	Long time consuming	Medical image
	[81]	Combined with band feature extraction algorithm	Band feature extraction algorithm	High search efficiency	Easy to fall into local optimization	Hyperspectral image
	[82]	Combined with least squares support vector machine	Least squares support vector machine	Recognition model enhancement	Increase the complexity of the algorithm	Green pepper target recognition
	[83]	Combined with Niblack algorithm	Niblack algorithm	Improved segmentation accuracy	Low efficiency	Infrared image diagnosis of power equipment
	[84]	Combining RBF neural network, support vector machine and AdaBoost	RBF neural network, support vector machine and AdaBoost	Reduce the correlation between features and improve the classification speed	Low recognition accuracy	Tobacco image features
SALP	[132]	Improve SALP algorithm	Genetic algorithm	Improve recognition efficiency	Long time consuming	Medical image
	[138]	Combined denary Salp Swarm Algorithm	Feature Selection	Speed up operation	Easy to fall into local optimization	Characteristic selection of converter steelmaking

**Table 5:** Comprehensive analysis of image edge detection.

	Algorithm	Briefly Analyze	Image Segmentation	Advantage	Disadvantage	Applicable Field
ACO	[51]	Combined with genetic algorithm	Genetic algorithm	Improve image edge detection quality	Easy to fall into local optimization	Medical CT image
	[52]	Incorporating pheromones for edge detection	Pheromone edge detection	Greatly reduce image blur	Long time consuming	UAV image target edge detection
	[53]	New DNA-ACO algorithm	DNA-ACO algorithm	Shorten the search time and improve the search accuracy	The image edge definition is not high	Medical image
	[54]	Combining gray gradient and regional gray mean method	Gray gradient and gray area mean method	Reduce the influence of noise on edge detection	Low efficiency	Infrared image
	[55]	Combined with ABC algorithm, maximum interclass variance	ABC algorithm, maximum interclass variance	Obtained edge information has better integrity	Edge detail detection is not ideal	Medical liver image
PSO	[85]	Improved optimal entropy threshold segmentation	Optimal entropy threshold segmentation technology	Better edge detection	Edge detection is slow	Medical image
	[86]	Introduction to quaternion image edge detection	Vector rotation principle of Quaternion	Extract image texture details, the algorithm is stable	Easy to fall into local optimization	Color image, texture image
	[87]	Linear matrix inequality combined with PSO to train cellular neural network	Cellular neural network	Overcome the shortcomings of the algorithm’s unsatisfactory edge detail detection	Algorithm is not stable	Medical image
	[88]	Improved PSO to optimize gradient operator	Gradient operator	Solve the problem of detail edge loss	Easy to fall into local optimization	Infrared image
	[89]	Combined with gray wolf algorithm and XOR encoding	Gray wolf algorithm, combined with XOR encoding	Improved accuracy for segmenting complex images	Long segmentation time	Asteroid surface image segmentation
BA	[120]	Combined Canny operator	Canny operator	Extracting edge information of defects	Slow edge detection	Medical brain image

## Data Availability

Not applicable.
